# Effect of parsley (*Petroselinum crispum*, Apiaceae) juice against cadmium neurotoxicity in albino mice (*Mus Musculus*)

**DOI:** 10.1186/s12993-016-0090-3

**Published:** 2016-02-04

**Authors:** Saleh N. Maodaa, Ahmed A. Allam, Jamaan Ajarem, Mostafa A. Abdel-Maksoud, Gadah I. Al-Basher, Zun Yao Wang

**Affiliations:** 1 Department of Zoology, College of Science, King Saud University, Riyadh, 11451 Saudi Arabia; 2 Department of Zoology, Faculty of Science, Beni-Suef University, Beni-Suef, Egypt; 3 State Key Laboratory of Pollution Control and Resources Reuse, School of the Environment, Nanjing University, Nanjing, 210023 Jiangsu People’s Republic of China

**Keywords:** Heavy metal, Cerebellum, Cerebrum, Medulla oblongata, Neurotransmitter

## Abstract

**Background:**

Parsley was employed as an experimental probe to prevent the behavioral, biochemical and morphological changes in the brain tissue of the albino mice following chronic cadmium (Cd) administration.

**Methods:**

Non-anesthetized adult male mice were given parsley juice (*Petroselinum crispum*, Apiaceae) daily by gastric intubation at doses of 10 and 20 g/kg/day. The animals were divided into six groups: Group A, mice were exposed to saline; Groups B and C, were given low and high doses of parsley juice, respectively; Group D, mice were exposed to Cd; Groups E and F, were exposed to Cd and concomitantly given low and high doses of parsley, respectively.

**Results:**

Cd intoxication can cause behavioral abnormalities, biochemical and histopathological disturbances in treated mice. Parsley juice has significantly improved the Cd-associated behavioral changes, reduced the elevation of lipid peroxidation and normalized the Cd effect on reduced glutathione and peroxidase activities in the brain of treated mice. Histological data have supported these foundations whereas Cd treatment has induced neuronal degeneration, chromatolysis and pyknosis in the cerebrum, cerebellum and medulla oblongata.

**Conclusion:**

The low dose (5 g/kg/day) of parsley exhibited beneficial effects in reducing the deleterious changes associated with Cd treatment on the behavior, neurotransmitters level, oxidative stress and brain neurons of the Cd-treated mice.

## Background

Cadmium (Cd) is among the most hazardous heavy metals that is not chemically degraded in the environment and can enter into the food chain [1]. Both natural and anthropogenic sources of this heavy metal, including industrial emissions and the application of fertilizer and sewage sludge to farm land, may lead to the contamination of soils and the increased Cd uptake by crops and vegetables grown for human consumption [2]. Some important sources of Cd exposure for humans can be the emissions from industries of petroleum mining, batteries, metal plating, pigments, plastics, toys and alloy, cigarette smoking and through dietary consumption [3]. Total human intake of Cd from food has been estimated by Järup [4] as 2.8–4.2 µg/kg body weight/week, which equates to approximately 40–60 % of the current provisional tolerable weekly intake of 7 µg/kg body weight/week.

Cd has no essential biochemical roles, but can exert diverse and severe toxicity in multiple body systems as it can bind to tissues, trigger oxidative stress, affect endocrine function, block aquaporins, and interfere with functions of essential cations such as magnesium and zinc [5]. Cd can pose some particular risks to the young, as exposures in early life can compromise development, with lifelong physical, intellectual, and behavioral impairments [6]. The International Agency for Research on Cancer classified cadmium as a well-known carcinogen [7]. Cd represented one of the most toxic and carcinogenic heavy metals [8]. It is considered as a serious health hazard to humans and other animals [9]. Exposure to Cd may cause lesions in many organs such as brain, liver, kidney and testis [10], thus leading to cerebral, hepatic, renal and testicular dysfunction [11]. It has been reported that Cd exposure produced long-term impairments of neurobehavioral status such as alterations in attention and memory as well as in the psychomotor and visuomotor functioning and speed in workers [12, 13]. Clinical data have shown aggressive elevation and anxiety-like behaviors, impaired learning and memory processes, and changes in the development of the visual system [14]. Previous studies on Cd toxicity have reported behavioral impairments in both animal models and humans after exposure to Cd [15]. Acetylcholine esterase is an important enzyme that hydrolyses the neurotransmitter acetylcholine in the synaptic cleft of cholinergic synapses and neuromuscular junctions [16]. Alterations in the acetylcholine activity in various diseases and poisonings suggested that this enzyme could be an important physiological and pathological biomarker [17, 18].

Additionally, Cd can increase blood–brain barrier permeability, thus penetrating and accumulating in brain tissue of animals [17] and leading to brain intracellular accumulation, cellular dysfunction, and cerebral edema. Also, it can affect the degree and balance of excitation–inhibition in synaptic neurotransmission as well as the antioxidant levels in animal’s brain [19, 20]. Cd toxicity is partly due to oxidative DNA damage associated with the increased production of reactive oxygen species (ROS), such as superoxide ion, hydroxyl radicals and hydrogen peroxide [21]. Previous studies have indicated that Cd can decrease antioxidant enzyme levels [21]. Free radicals cause the oxidation of biomolecules (e.g., protein, amino acids, lipid and DNA), which leads to cell injury and death [22]. For example, ROS markedly alter the physical, chemical, and immunological properties of superoxide dismutase (SOD), which further exacerbates oxidative damage in cells. This has raised the possibility that antioxidants could acts as prophylactic agents against many pathological conditions. It has long been recognized that naturally occurring substances in higher plants have antioxidant activities. Many culinary herbs (e.g., parsley) have been shown to function as natural antioxidants [23].

Parsley (*Petroselinum crispum*, Apiaceae) is an annual herb, which is important dietary source of vitamins and essential metals. It’s usage at sufficient levels whether cooked or not cooked can promote the levels of vitamins and essential metals in human body, which in turn can decrease the risks of Cd toxicity [24]. Phytochemical screening of parsley has revealed the presence of some compounds such as flavonoids [25], carotenoids [26], ascorbic acid [27] and tocopherol [28]. These components of fresh parsley leaf can scavenge superoxide anion in vitro and hydroxyl radical in addition to protecting against ascorbic acid-induced membrane oxidation [25]. Supplementation of diets with fresh parsley leaf can increase antioxidant capacity of rat plasma and decrease oxidative stress in humans [29]. Similarly, aqueous and ethanol extracts of fresh parsley leaf strongly inhibit linoleic acid oxidation and lipid oxidation [30]. Biological mobility, tissue concentrations, and excretion of Cd are determined by oxidation state, solubility, a complex set of equilibria between complexing sites, as well as active transport through membranes [31]. Chelation is central to natural detoxification of heavy metals, via formation of complexes, particularly with glutathione and other small molecules and their excretion [7]. Essential oil extracted from parsley possessed a certain degree of antioxidant activities in terms of β-carotene bleaching capacity and free radical scavenging activity [32]. Therefore, parsley was also reported as a possible source of antioxidants which may prevent Cd toxicity.

Also, parsley is one of the most used medicinal plants to treat arterial hypertension [33]; diabetes, cardiac [33] and renal diseases [34]. Moreover, in experimental studies, it has been reported that this herb has strong diuretic [35], anti-hyperglycemic, anti-hyperlipidemic, anticoagulant [36], anti-oxidant [32], anti-microbial [37] and laxative activities [38]. It has been reported that parsley alcoholic extract has a protective effect against toxicity induced by sodium valproate in male rats [39]. Parsley leaves were used for treatment of constipation, jaundice, colic, flatulence edema, rheumatism. It was used to treat eczema, knee, ache, impotence and bleeding [40]. However, according to our knowledge, the reported literatures on the protective effect of parsley against Cd neurotoxicity are still limited.

In the present work, we investigated the hypothesis that parsley juice may protect against Cd-induced pathological changes in albino mice.

## Methods

### Chemicals

Cadmium chloride (CdCl_2_) was of analytical grade and purchased from Sigma Chemical Company (St Louis, MO, USA).

### Parsley juice preparation

*petroselinum crispum* (*mill.*) *nymex a.w. hill* from the family Apiaceae (alt. Umbelliferae) is commonly known as parsley. The origin of parsley is from Mediterranean region, but today is cultivated wherever of the world. Botanic identification was performed by taxonomist in the Department of botany and microbiology, Faculty of Sciences, King Saud University (Riyadh, Saudi Arabia) where a voucher specimen has been deposited (collection number AL 1021). The plain leaf parsley type daily collected from vegetable market in Riyadh (Saudi Arabia) was carefully washed under tap water. The fresh parsley juice was prepared daily using a vegetable juicer. Two concentrations of the juice were prepared: the first is 10 % juice, i.e., 10 g parsley squash in 100 ml drinking water. The second is 5 % juice, i.e., 5 g parsley squash in 100 ml drinking water. The prepared juice has been filtered using a filter paper after preparation and before drinking by the animals to remove fibers and other insoluble components.

### Ethics statement

All the experimental protocols and investigations were approved and complied with the *Guide for Care and Use of Laboratory Animals* published by the US National Institutes of Health (NIH Publication No. 85–23, revised 1996) and was approved by the Ethics Committee for Animal Experimentation at King Saud University (Permit Number: PT 983).

### Animals and dosing schedule

A total of 48 adult male albino mice (*Mus musculus*) weighing 30–35 g were obtained from College of Pharmacy, King Saud University, Saudi Arabia and housed in stainless steel wire cages (5 animals/cage) under specific pathogen-free conditions. The animals were maintained at 22–25 °C on a 12:12 h light/dark cycle and provided with food and water ad libitum. Parsley juice was orally administered daily to non-anesthetized parsley treated groups by gastric intubation at two doses of 10 and 20 g/kg/day for 28 days (D). Totally, 30 mg/kg of CdCl_2_ dissolved in saline was intraperitoneally injected to partially anesthetized Cd treated groups by three exposure times D1, D7 and D15 (10 mg/kg every time). The animals were labeled into six groups as follows:

- Group A: mice were given with tab water orally and saline intraperitoneally (*Control group*).
- Group B: mice were given with 5 % Parsley juice orally and saline intraperitoneally (5 % *Parsley group*).
- Group C: mice were given with 10 % Parsley juice orally and saline intraperitoneally (10 % *Parsley group*).
- Group D: mice were given with tab water orally and Cd doses intraperitoneally (*Cd intoxicated group*).
- Group E: mice were given with 5 % Parsley juice orally and Cd doses intraperitoneally (5 % *Parsley*-*Cd group*).
- Group F: mice were given with 10 % Parsley juice orally and Cd doses intraperitoneally (10 % *Parsley*-*Cd group*).

### Cd estimation assay

#### Instrumentation

The analytical determination of Cd in mice brain was carried out by ICP-MS (inductively coupled plasma mass spectrometer): ELAN 9000 (Perkin Elmer Sciex Instrumento, Concord, Ontario, Canada).

#### Reagents

Nitric acid (69 % v/v), super purity grade was supplied from Romil, England. Hydrochloric acid (37 % v/v) and hydrofluoric acid (40 % v/v) were supplied from Merck (Germany). High purity water obtained from Millipore Milli-Q water purification system was used throughout the work.

#### Calibration

The ICP-MS calibration was carried out by external calibration with the blank solution and three working standard solutions (20, 40 and 60 ppm), starting from 1000 mg/l single standard solutions for ICP-MS (A ristar grade, BDH laboratory supplies, England for Cd).

#### Sample collection and preparation

Samples were prepared by accurately weighing 200 mg of longitudinal section of brain into a dry and clean Teflon digestion beaker, 6 ml of HNO_3_, 2 ml HCl and 2 ml HF were added to the Teflon beaker. Samples were digested on the hot plate at 120–150 °C for approximately 40 min. The resulting digest was not clear, so it was filtered through whatman filtered paper No. 42. The filtered digest was transferred to a 50 ml plastic volumetric flask and made up to mark using deionized water. A blank digest was carried out in the same way.

### Behavioral assays

8 male mice from each group were used in the present study at the beginning of the 5th week (D28–30) after finishing the dosing period. The animals were used only in one test of the present tests per day. For the tests, the animals were brought in a room (25 °C) of dim red light reserved for that purpose. All tests were conducted blindly by the same experimenter [41].

### T-maze conducting assay

The six groups of animals were prevented from food all the night before this examination. The elevated T-maze consisted of three closed arms to be T like structure. The main arm (100 × 10 × 20 cm) and two lateral arms (40 × 10 × 20 cm) at an elevation of 20 cm above the floor. Arms of the maze form T like structure. The rodent food was placed at the end of the right lateral arm. The maze was cleaned with a 20 % ethanol after each test. The hungry animals were placed in the terminal end of the main arm of the elevated T-maze facing the passage to the two lateral arms. The mice left to explore the maze for 1 min, then the animal removed from the maze and kept in its cage for 2 h. The mice replaced in the same position in the main arm and the behavior analyzed for 5 min by an experimenter who is blind for the experimental protocol. The frequency and duration in food and main arm visits (under red illumination) were recorded. The time spent exploring the arms to reach food (seconds), and the time spent in food arm in seconds, were determined. The frequency and time of entering the food lateral arm not the other lateral arm was considered to be memory reflector according to Leret [42].

#### Cage activity assay

The Ugo Basile 47420-Activity Cage was used to record spontaneous co-ordinate activity of mice and variation of this activity in time either horizontal or vertical movements. This test was performed for 3 min/animal.

#### Grip-strength meter assay

The Ugo Basile 47200-Grip-Strength Meter suitable for mice automatically measures grip-strength (i.e. peak force and time resistance) of forelimbs in mice. The aim was to assess forelimbs muscle strength. Each animal was tested for three times and the peak force of each mouse was recorded. The mean of three values of each mouse was recorded.

#### Rota-rod assay

The Ugo Basile rota-rod instrument was used in this test, the mouse is placed on a horizontally oriented and mechanically rotating at 15 rpm rod. The rod is suspended above a cage floor, which is low enough not to injure the animal, but high enough to induce avoidance of fall. Mice naturally try to stay on the rotating rod, or rota-rods, and avoid falling to the ground. The length of time that a given animal stays on this rotating rod is a measurement of their balance, coordination, physical condition, and motor-activity.

### Biochemical assays

8 animals of each group were anesthetized by light ether and sacrificed at D30. Brain was dissected and 0.5 g tissue was homogenized in 5 ml of cold 0.1 M HClO_4_ containing 0.05 % EDTA. The homogenate was centrifuged at 10,000 rpm for 10 min at 4 °C and the clear supernatant collected in a microfuge tube (0.5 ml each) and stored at −40 °C until assays.

#### Dopamine and serotonin determination

The level of neurotransmitters 5-hydroxytryptamine or serotonin and dopamine was estimated in the brain. The monoamines dopamine and serotonin was estimated using the modified method of Patrick [43]. A 10 % homogenate of the brain has been re-centrifuged at 17,000 rpm at 4 °C for 5 min. The supernatants were filtered using 0.45 μm pore filters and analyzed by high performance liquid chromatography. The mobile phase consisted of 32 mM citric acid monohydrate, 12.5 mM disodium hydrogen orthophosphate, 7 % methanol, 1 mM octane sulfonic acid and 0.05 mM EDTA. The mobile phase was filtered through 0.22 μm filter and degassed under vacuum before use. Bondpak C_18_ column was used at a flow rate of 1.2 ml/min and the injection volume of the sample was 20 μl. The levels of dopamine and serotonin were calculated using a calibration curve and the results were expressed as ng/mg tissue weight.

#### Determination of acetylcholine

The method has been described previously which didn’t use acetylcholine esterase inhibitor [44]. Inbrief, dialysate samples were directly injected into the liquid chromatography/electrochemistry system assisted by a chromatography manager (Millennium; Waters, Milford, MA) and analyzed for acetylcholine. Acetylcholine was separated on a coiled cation exchanger acetylcholine column (analytical column) (Sepstik 530 × 1.0 mm I.D., packed with polymetric strong exchanger, 10 μm in diameter; BAS, West Lafayette, IN), followed by the post-immobilized enzyme reactor which consisted of choline oxidase/acetylcholine esterase. Acetylcholine was hydrolyzed by acetylcholine esterase to form acetate and choline in the post-immobilized enzyme reactor, and then choline was oxidized by choline oxidase to produce betaine and hydrogen peroxide (H_2_O_2_). H_2_O_2_ is detected via oxidation of horseradish peroxidase, which in turn oxidized Os (bpy) entrapped in the redox polymer coated on the surface of the glassy carbon electrode (MF-9080; BAS), set at +100 mV (LC-4C; BAS) versus Ag/AgCl reference electrode. This reduction was analyzed with the detector (LC-4C; BAS) as a signal indicating acetylcholine on the chromatogram.

#### Lipid peroxidation assay (TBARS)

Lipid peroxidation was determined by assaying thiobarbituric acid-reactive substances (TBARS) according to the method of Preuss [45]. Briefly, 1.0 ml supernatant was precipitated with 2 ml 7.5 % trichloroacetic acid and centrifuged at 1000*g* for 10 min. Clear supernatant was mixed with 1 ml 0.70 % thiobarbituric acid, incubated at 80 °C and the absorbance measured at 532 nm. Tetramethoxypropane was used as the standard.

#### Glutathione (GSH) assay

Glutathione content was determined according to the procedure of Beutler [46] with some modifications. Briefly, 0.20 ml of tissue supernatant was mixed with 1.5 ml precipitating solution containing 1.67 % glacial metaphosphoric acid, 0.20 % Na-EDTA and 30 % NaCl. The mixture was allowed to stand for 5 min at room temperature and centrifuged at 1000*g* for 5 min. One ml clear supernatant was mixed with 4 ml 0.30 M Na_2_HPO_4_ and 0.50 ml DTNB reagent (40 mg 5, 5′ dithiobis-(2-nitrobenzoic acid dissolved in 1 % sodium citrate). A blank was similarly prepared in which 0.20 ml water was used instead of the brain supernatant. The absorbance of the color was measured at 412 nm in a spectrophotometer.

#### Peroxidase activity determination

Peroxidase activity was determined according to the method of Kar and Mishra [47]. Briefly, 1.0 ml supernatant was mixed with 3.0 ml of 0.01 M phosphate- buffered saline (pH 6.8), 315 μl of 2 % pyrogallol, 154 μl H_2_O_2_ and incubated for 15 min at 25 °C. The reaction was stopped by the addition of 0.50 ml of 5 % H_2_SO_4_ and the absorbance was recorded at 420 nm. Peroxidase activity was expressed as the amount of purpurogallin formed per unit absorbance.

### Histological preparations

For the histological preparations, left loop of cerebellum, cerebral cortex and medulla oblongata of three sacrificed animals were immediately fixed in 20 % formalin saline for 24 h. The tissues were washed to remove the excessive fixative and then dehydrated in ascending grades (70, 80, 90 and 95 %) of ethyl alcohol for 45 min each, then in two changes of absolute ethyl alcohol for 30 min each. This was followed by two changes of xylene for 30 min each. The tissues were then impregnated with paraplast plus (three changes) at 60 °C for 3 h and then embedded in paraplast plus. Sections (4–5 µm) were prepared with a microtome, de-waxed, hydrated and stained in Mayer’s haemalum solution for 3 min. The sections were stained in Eosin for one min, washed in tap water and dehydrated in ethanol as described above. Hematoxylin and eosin (H&E) stained sections were prepared according to the method of Mallory [48].

### Statistical analysis

The Statistical Package for the Social Sciences (SPSS for windows version 11.0; SPSS Inc, Chicago) was used for the statistical analyses. Comparative analyses were conducted by using the general linear models procedure (SPSS, Inc). Also, the data were analyzed using one-way and two-way analysis of variance (ANOVA) followed by LSD computations to compare various groups with each other. Results were expressed as mean ± S.D. The level of significance was expressed as significant at P < 0.05, highly significant at P < 0.01 and very highly significant at P < 0.001.

## Results

### Cd bioaccumulation

**Fig. 1:**
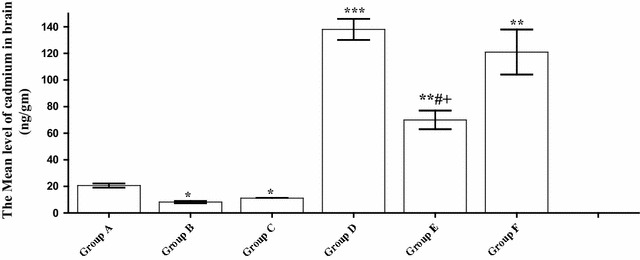
The mean concentration of Cd in the brain tissue of Cd-treated animals in comparison to control groups. Data are expressed as mean ± SEM (N = 8) (F = 28.446). *P < 0.05 for parsley (Groups B and C), Cd (Group D) and parsley + Cd treated groups (Groups E and F) versus control (Group A); ^#^P < 0.05 for parsley + Cd treated groups versus cadmium group; ^+^P < 0.05 for low dose parsley treated group versus high dose parsley treated group. (**P < 0.01; ***P < 0.001)

Cd concentrations in the brain tissues of exposed mice were estimated. As shown in Fig. 1, Cd bioaccumulation in the brain of both parsley groups (Groups B and C) was significantly (P < 0.05) reduced in comparison to the control group. On the other hand, a highly significant (P < 0.001) increase in the level of Cd in Cd-intoxicated group (Group D) in comparison to the control group, was monitored. Meanwhile, parsley treatment has an obvious ameliorating effect on Cd-intoxication. Both the low (5 %) and the high (10 %) doses of parsley have clearly decreased the Cd level in the brain tissues of Cd-parsley groups in comparison to the Cd-group (Group D) with the more reducing effect being associated with the low parsley dose (Group E).

### Effect of parsley treatment on the body weight change in Cd- treated mice

Toxicological studies have illustrated that many toxicants are usually associated with weight loss in exposed animals. In the current study, we have investigated the dampening effect of Cd toxicity on the body weight increase in treated mice and the possible restoring capacity of parsley on this effect. Table 1 illustrates that both parsley groups (B and C) have exhibited a similar pattern of body weight change in comparison to the control group (A) during the experiment time. Conversely, Cd-intoxicated group (D) has showed a significant decrease in the body weight in comparison to the control group. Parsley treatment has restored to some extent, the normal pattern of body weight change seen in the control groups.

**Table 1:** Effect of parsley treatment on the animal’s body weight change

	Initial weight	Final weight
Group A	32.21 ± 3.818	35.8 ± 4.131
Group B	32.87 ± 4.079	35.122 ± 5.638
Group C	32.81 ± 5.178	33.72 ± 5.5
Group D	33.7 ± 6.549	32.9 ± 7.1*
Group E	31.2 ± 4.589	33.97 ± 4.095^#^
Group F	31.8 ± 4.131	32.2 ± 6.71^#^

Initial and final body weights were estimated for all the five groups of animals. Data are expressed as mean ± SEM (N = 8). *P < 0.05, **P < 0.01, ***P < 0.001, for parsley (Groups B and C), Cd (Group D) and parsley + Cd treated groups (Groups E and F) versus control (Group A); ^#^P < 0.05 for parsley + Cd treated groups versus cadmium group; ^+^P < 0.05 for low dose parsley treated group versus high dose parsley treated group

### Behavioral investigations

**Fig. 2:**
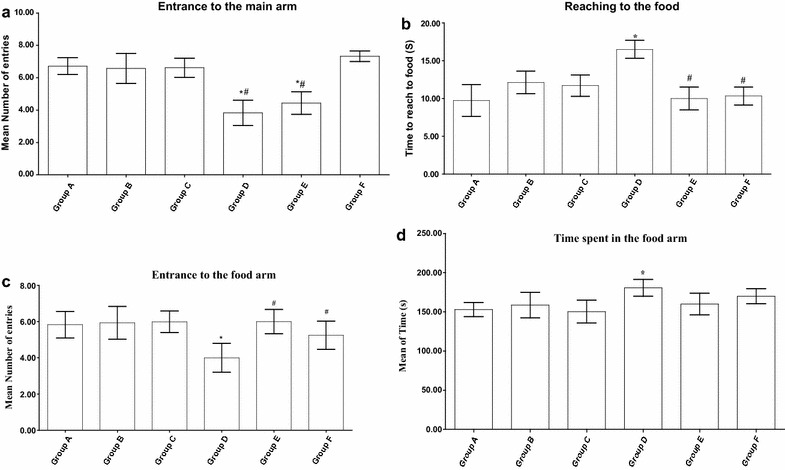
Parsley treatment can affect animal’s behavior. Animal’s behavior in T-maze. **a** The number of entrances of main arm (F = 3.448). **b** The time consumed to reach to the food (F = 8.076). **c** The number of entrances to the food arm (F = 3.471). **d** The time spent in the food arm (F = 8.780). Data are expressed as mean ± SEM (N = 8). *P < 0.05 for parsley (Groups B and C), Cd (Group D) and parsley + Cd treated groups (Groups E and F) versus control (Group A); ^#^P < 0.05 for parsley + Cd treated groups versus cadmium group; ^+^P < 0.05 for low dose parsley treated group versus high dose parsley treated group

**Fig. 3:**
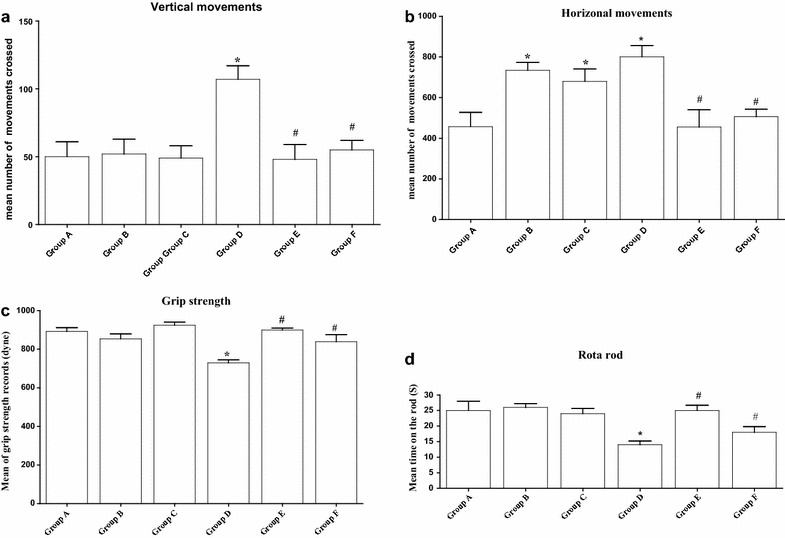
Effect of parsley treatment on the animal’s behavior. **a**–**d** The vertical movements, horizontal movements, the fore-limb grip strength records and rota-rod records for the animals of each group (F = 11.282, F = 10.52, F = 40.77, F = 3.74), respectively. Data are expressed as mean ± SEM (N = 8). *P < 0.05 for parsley (Groups B and C), Cd (Group D) and parsley + Cd treated groups (Groups E and F) versus control (Group A); ^#^P < 0.05 for parsley + Cd treated groups versus cadmium group; ^+^P < 0.05 for low dose parsley treated group versus high dose parsley treated group

The behavior of animals in the T-maze showed that the Cd treated animals have bad memory and low smell ability. This was elucidated by the decreased number of entrances to the main arm (Fig. 2a) and to the food arm (Fig. 2c) concomitant with the long time consumed by the Cd-treated animals to reach to the food (Fig. 2b) and to the food arm (Fig. 2d) in comparison to the control animals. Parsley treatment has showed an ameliorating effect that was clearer in the low dose parsley group in comparison to the high dose one. In the activity cage, the animals of Cd-intoxicated group showed a significant elevation in the vertical and horizontal movements as compared to the control group. On the contrary of the vertical movement, the animals of both parsley groups (Groups B and C) showed significant increases in the values of horizontal movements. After parsley treatment, the animals of Groups E and F displayed values of vertical and horizontal movements that were near to that of the control group animals (Fig. 3a, b).

The fore-limb muscles of mice in Groups A, B and C recorded relatively similar beaks in the grip strength examination scores. The recorded beaks of Group D animals appeared significantly lower in comparison to the control, the beaks achieved by the animals of Groups E and F showed significant improvements (Fig. 3c).

In rota-rod test and in comparison to the control group, the latency to fall by the Cd-intoxicated group on the rod was significantly reduced. Parsley treatment has resulted in increasing the staying times of the animals of Groups E and F on the rod to become near to that of the control group (Fig. 3d).

### Biochemical studies

#### Neurotransmitters

**Fig. 4:**
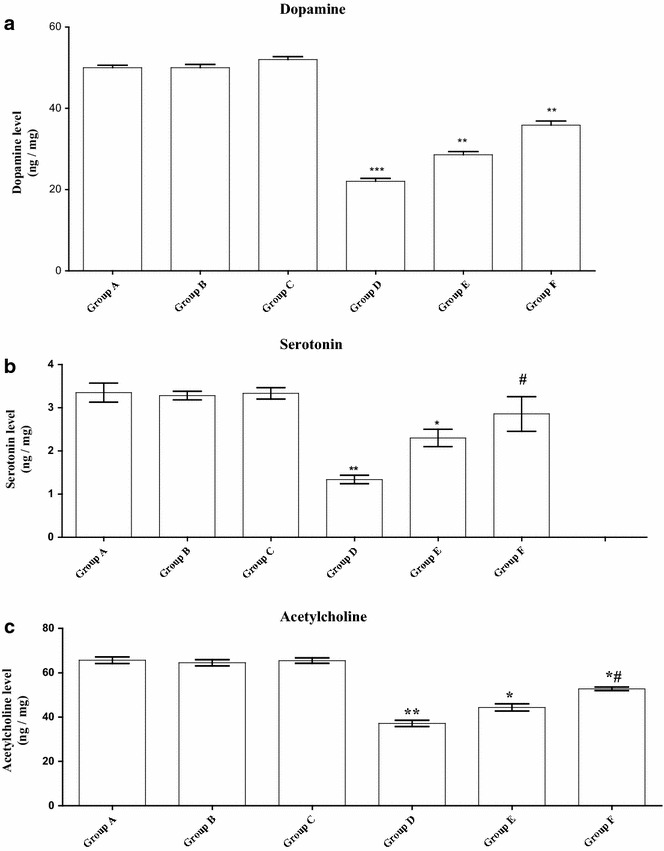
Effect of parsley treatment on the brain level of neurotransmitters. **a**–**c** The mean concentration of extra cellular dopamine, serotonin and acetylcholine in the animals brain tissues of each group (F = 249.63, F = 31.24, F = 169.79), respectively. Data are expressed as mean ± SEM (N = 8). *P < 0.05, **P < 0.01,***P < 0.001, for parsley (Groups B and C), Cd (Group D) and parsley + Cd treated groups (Groups E and F) versus control (Group A); ^#^P < 0.05 for parsley + Cd treated groups versus cadmium group; ^+^P < 0.05 for low dose parsley treated group versus high dose parsley treated group

Dopamine is an important neurotransmitter and its concentration is usually linked with the functioning of the nervous system and the behavior of living organisms. In both of the control and the parsley groups (A, B and C), the dopamine level in mice brain appeared nearly similar with little elevation in Group C. In comparison to the control group, a highly significant depletion of dopamine concentration has been detected in Cd-intoxicated group (P < 0.001). Parsley treatment has slightly ameliorated this effect but still, a significant reduction in Groups E and F (P < 0.01)was recorded (Fig. 4a). Serotonin level, another important neurotransmitter, was investigated in all the experimental groups. Both parsley groups (B and C) have showed no significant difference in the brain level of serotonin in comparison to the control group. A significant reduction in the level of serotonin was detected in Cd-intoxicated group (D). Following parsley treatment with two different doses, the level of serotonin was significantly reduced in the low dose parsley group (E) while the high dose one (F) has showed no significant reduction as compared to the control (Fig. 4b).

The present acetylcholine concentrations showed similar results to dopamine. In Groups A, B and C, its concentration did not show any significant difference, while it displayed a highly significant (P < 0.01) depletion in Cd-intoxicated group. In Groups E and F, a significant (P < 0.05) reduction in serotonin level was also monitored but still, parsley treatment has an obvious lowering effect on the Cd-induced pathology (Fig. 4c).

#### Oxidative stress

**Fig. 5:**
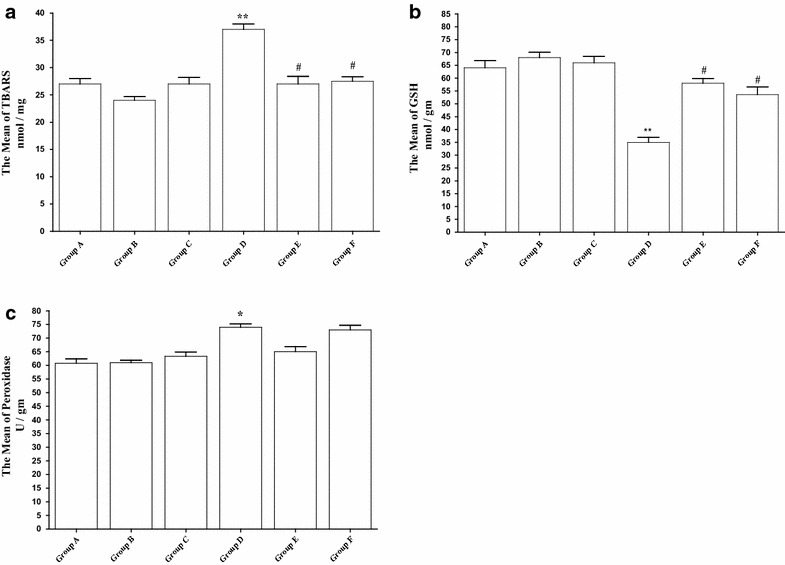
Effect of parsley treatment on Cd-induced oxidative stress. **a**–**c** The mean concentration of TBARS, GSH and peroxidase in the brain tissues of each group (F = 124.53, F = 189.94, F = 154.65), respectively. Data are expressed as mean ± SEM (N = 8). *P < 0.05, **P < 0.01, ***P < 0.001, for parsley (Groups B and C), Cd (Group D) and parsley + Cd treated groups versus control (Groups E and F); ^#^P < 0.05 for parsley + Cd treated groups versus cadmium group; ^+^P < 0.05 for low dose parsley treated group versus high dose parsley treated group

As illustrated in Fig. 5a, the change in brain lipid peroxidation in parsley groups was not significant in comparison to the control group. Conversely, a significant (P < 0.01) elevation in TBARS level was detected in Cd-intoxicated group. However, after parsley treatment the TBARS level was significantly lowered in both Cd-parsley groups (Groups E and F) in comparison to Cd group (Group D).

In addition to the increased level of TBARS, Cd treatment has also produced a significant (P < 0.001) decrease in GSH content in Cd-intoxicated group. Parsley has exerted a positive impact and improvement was observed in both of parsley-Cd groups as indicated from the significant increase in Groups E and F in comparison to the Cd group (D) (Fig. 5b).

Peroxidase activity was also monitored as an important indicator for oxidative stress. While both parsley groups have a similar level of peroxidase in comparison to the control, Cd-intoxicated group has showed a significant elevation in the peroxidase activity as compared to the control (Fig. 5c). After parsley treatment, both parsley-Cd groups have showed decreased level of peroxidase as compared to the Cd group.

### Brain histopathological changes

**Fig. 6:**
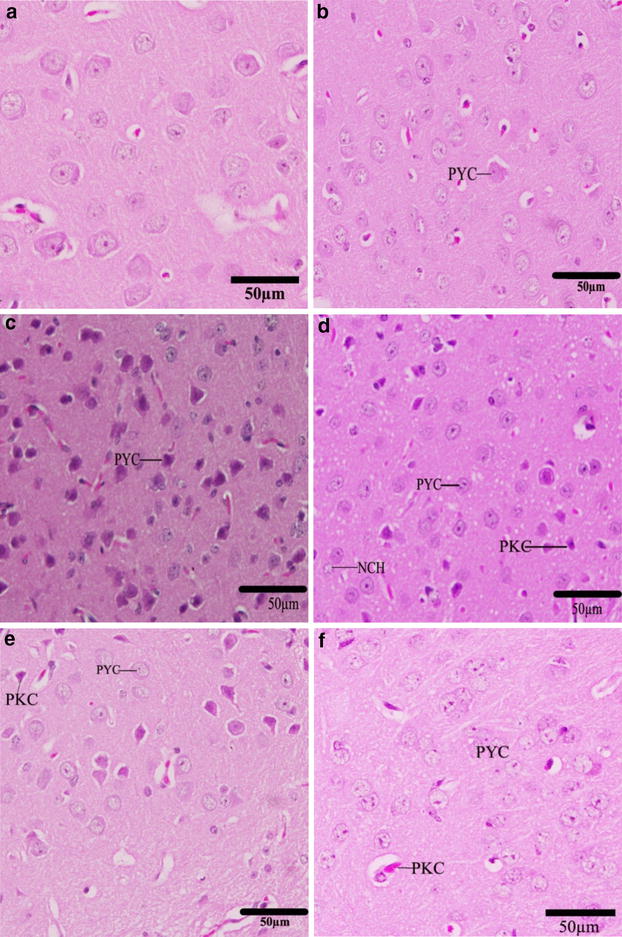
Effect of parsley treatment on the histology of cerebral cortex. Photographs of the cerebral cortex of the newborns at D30 showing pyramidal neurons (PYC) degenerated pyramidal cells (PKC), neurocytechromatolysis (NCH). **a** Control group, **b** Parsley 5 % group (Group B), **c** Parsley 10 % group (Group C), **d** Cd-group (Group D), **e** Cd + 5 % parsley group (Group E) and **f** Cd + 10 % parsley group (Group F) (H & E stain)

The normal pyramidal neurons exhibited their general characteristic shape. The nuclei of these cells were rounded, large and centrally located (Fig. 6). The normal cells of the cerebral cortex had spherical or pyramidal perikaryon, whose nuclei were large; also the neurons were arranged in a regular pattern (Fig. 6a–c). The cerebral neurons appeared more developed toward the white matter. In Cd-treated group, chromatolysis and pyknosis have been observed in the pyramidal neurons. In Groups E and F, parsley juice showed significant neuronal protection through reducing the rate of chromatolysis and pyknosis (Fig. 6d–f).

**Fig. 7:**
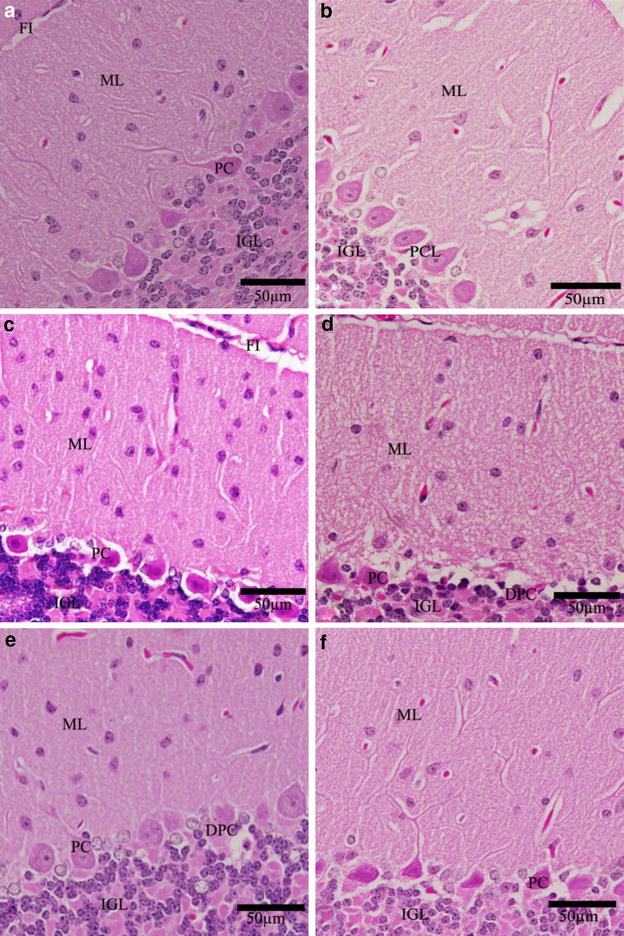
Effect of parsley treatment on the histology of cerebellar cortex. Photographs of the cerebellar cortex of the newborns at D30 showing Purkinje cell (PC),Purkinje cell layer (PCL), degenerated Purkinje cell (DPC), fissure (FI), hemorrhage (H), internal granular layer (IGL), molecular layer (ML) and white matter (WM). **a** Control group, **b** parsley 5 % group (Group B), **c** parsley 10 % group (Group C), **d** Cd-group (Group D), **e** Cd + 5 % parsley group (Group E) and **f** Cd + 10 % parsley group (Group F) (H & E stain)

In the cerebellum, the fold layers (molecular, Purkinje cells and internal granular) became completely mature and the external granular layer disappeared completely (Fig. 7). The neuronal density in the molecular layer of both the control and the parsley treated groups was the highest as compared to Cd-treated groups. The normal Purkinje cells were arranged in a single row of large neurons with pear-shaped perikaryon and large nucleus. The lateral processes were disappeared and the apical processes formed the permanent dendritic tree (Fig. 7). In Cd-treated groups, some degenerated and pyknotic Purkinje cells were detected. Of which, some were more spindle-shaped and small. These numbers of degenerated Purkinje neurons were reduced in Cd-parsley groups (Groups E and F) (Fig. 7d–f). Variations have been observed in the folds size of the groups, where small folds appeared in Group D.

**Fig. 8:**
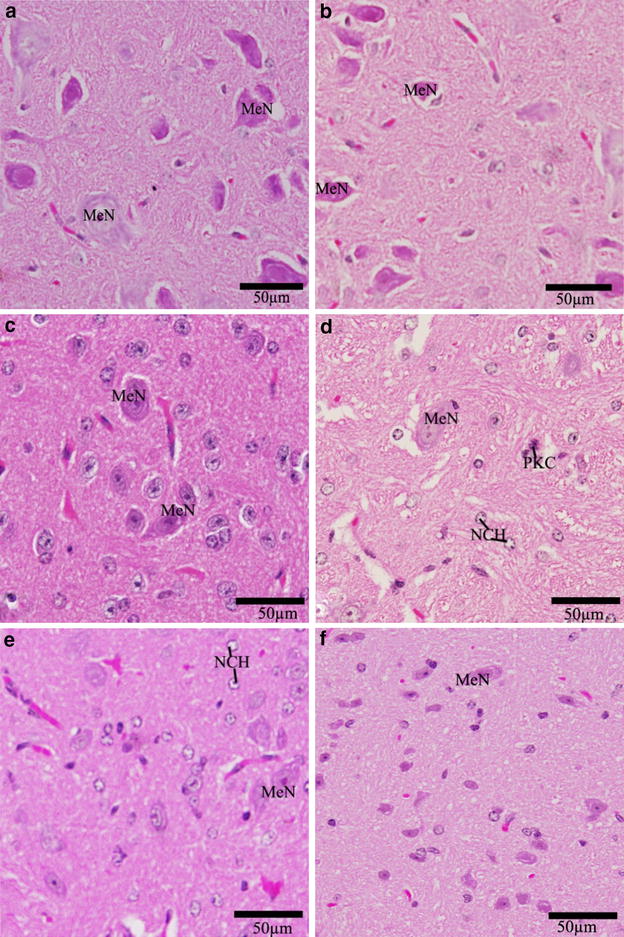
Effect of parsley treatment on the histology of medulla oblongata. Photographs of the medulla oblongata of the newborns at D30 showing medulla neurons (MeN), degenerated medullary cells (PKC), neurocytechromatolysis (NCH). **a** Control group, **b** parsley 5 % group (Group B), **c** parsley 10 % group (Group C), **d** Cd-group (Group D), **e** Cd + 5 % parsley group (Group E) and **f** Cd + 10 % parsley group (Group F) (H & E stain)

The normal medulla neurons appeared large in size, polygonal, varied in shape and had round nuclei (Fig. 8a–c). In Cd-treated group, most medulla neurons appeared small and pyknotic (Fig. 8d). In Cd-parsley treated groups, the medulla neurons showed improvement (Fig. 8e, f).

## Discussion

The present study was designed to investigate the protective role of parsley juice against Cd neurotoxicity in albino mice. The obtained results suggested that intake of parsley may partly improve the malformations induced by exposure of adult mice to Cd. The effects of daily supplementation of two different doses of parsley on the deleterious changes of Cd on animal’s behavioral activities, neurotransmitters level, oxidative stress parameters and histopathology of brain were investigated in the current study. The high concentration of Cd in brain tissues of Cd-treated groups may be due to the Cd bioaccumulation. The current results of Groups E and F showed that parsley juice has an observable protective effect against Cd accumulation especially at the low dose. This may be due to the significant effect of parsley in the excretion of heavy metals from body, an effect that was documented before [35]. In fact the dose dependence of parsley has been illustrated before [49] and it seems in the current study that the low parsley dose is considered as a therapeutic dose while the high dose one is considered as overdose. Previously, animal’s body weight was known to be one of the most sensitive indicators of toxicity [49]. In a recent study of Gonçalves [17], the rodents exposed to Cd were reported to display a lowered body weight. Our data supports these previous foundations in Cd-intoxicated group. However, in Cd-parsley groups, parsley could improve this effect and this may be attributed, in part, to the beneficial actions that parsley exerts on the gastrointestinal tract [51].

The results of the present study showed that the brain levels of neurotransmitters were significantly depleted by Cd treatment in Cd-exposed mice. It was reported previously that neurotransmitter depletion is nutritionally based. Neurotransmitters are made from amino acids that are required for its creation. Consequently, if the diet is deficient, neurotransmitter deficiency develops [52]. Here, Cd-intoxication may have been resulted in anorexia and consequently diet deficiency. There is an evidence of an inhibitory role of dopamine and serotonin mediated receptors in depressing the hyperexcitability of brain neurons as appeared by the poor performance of the treated animals in the current behavioral examinations [8]. Recently, many research groups have focused on the participation of serotonin in the neurochemical mechanisms of cognition, especially of learning and memory. Potential toxic mechanisms of action for Cd may include the disruption in serotonergic neurotransmission through disturbed levels of neurotransmitters in mice brain [50]. It was reported before that Cd toxicity has resulted in disrupted acetylcholine esterase activity [15]. Cd-intoxicated group of animals showed greater oxidative stress and a marked depletion of the antioxidants, than Groups E and F animals that were exposed to Cd and parsley juice. The results presented in the current study showing higher increase of the lipid peroxidation in Group D which is in accordance with the results obtained by Méndez-Armenta and Ríos [19] that reported an enhanced lipid peroxidation and increased TBARS after acute Cd exposure. Glutathione is one of the essential compounds for maintenance of cell integrity and participation in cellular metabolism [51]. Alterations in the ratio of reduced (GSH) and oxidized (GSSG) glutathione are well accepted as one of the indicators of oxidative stress in humans and experimental animals [52, 53].

The depletion of GSH may lead to lipid peroxidation. Therefore, GSH is considered as an important biomarker of oxidative stress [54]. The level of GSH is regulated by NADPH dependent enzyme, glutathione reductase (GR), and therefore an inhibition of GR may adversely affect GSH levels [54]. Furthermore, conjugation of heavy metals metabolites, with GSH forms glutathione-S-conjugates, which ultimately forms mercapturic acids [55]. This may further deplete GSH in the cell. Abu-Taweel [8] reported that Cd depleted GSH content and increased peroxidase enzymes activities in mice. Increased peroxidase activity might be due to generation of free radicals [56]. The antioxidant enzymes (e.g. peroxidases) constituted a mutually supportive team of defense against ROS [54].

Several mechanisms can contribute to the increased oxidative stress in toxicity induced by heavy metals, especially chronic exposure to Cd. Accumulated evidence pointed out that Cd inhalation can lead to elevated ROS and reactive nitrogen species (RNS) production by the mitochondrial respiratory system, antioxidant enzyme inactivation and an imbalance of glutathione redox status [13]. Cd toxicity can promote an important oxidative imbalance, favoring the production of free radicals and the reduction of antioxidant defenses. At high concentrations, ROS/RNS can damage the major components of the cellular structure, including nucleic acids, proteins, amino acids, and lipids [57]. Such oxidative modifications would affect several cell functions, metabolism, and gene expression, which in turn can cause some other pathological conditions [58]. The oxidative stress leads to neuronal damage in several brain regions [54–59]. For example, neuronal loss in the cerebrum can impair animal’s memory [60] while, neuronal loss in the cerebellum can have some adverse effects on balance and coordination [55]. In addition, neuronal loss in the medulla oblongata and the spinal cord can affect physical activity of mice [61]. Supplementation with parsley juice for 28 days alleviated somewhat the Cd-induced toxicity, which showed significant improvement in the physical balance, memory, coordination, motor activities, muscles strength and brain neurotransmitters levels in Cd-treated animals. Parsley supplementation also restored GSH balance and decreased lipid peroxidation and peroxidase activity. Overall, this study demonstrated that the low dose (10 g/kg/day) of parsley supplementation could improve the pathological alterations in mice and this is in accordance with the results reported by Zhang [32]. Parsley has also been found to significantly suppress hydroperoxide and ROS levels in brain and other tissues in mice by stimulating production of glutathione synthesis and thereby boosting cellular antioxidant defenses [32]. Taken together, our data demonstrates that parsley may be an important therapeutic tool to combat Cd toxicity-associated effects. This parsley ameliorating effect may be due to its ability to neutralize free radicals and thereby prevent neuronal damage caused by oxidative stress.

## Conclusion

Parsley has protective effects against Cd neurotoxicity in albino mice. Parsley juice supplementation improves the abnormal behavior of Cd intoxicated mice and reduces neuronal aberrations in the brain.

## Abbreviations

D: day
EDTA: ethylene diamine tetraacetic Acid
ICP-MS: inductively coupled plasma mass spectrometer
GSH: reduced glutathione
ROS: reactive oxygen species
RNS: reactive nitrogen species
TBARS: thiobarbituric acid-reactive substances
